# IgG4-RD-Associated Mikulicz Syndrome Without Classic Systemic Involvement—A Case Report

**DOI:** 10.3390/jcm14030958

**Published:** 2025-02-02

**Authors:** Luis Ángel Mendoza-Vargas, Samuel Sevilla-Fuentes, Brandon Bautista-Becerril, Bertha Berthaúd-González, Ramcés Falfán-Valencia, Linda P. Félix-Martínez, Mauricio Avila-Páez, Jennifer Manilla-González

**Affiliations:** 1 Hospital General de Zona 1 “Emilio Varela Luján”, Zacatecas 98000, Mexicosamuelsevilla2000@hotmail.com (S.S.-F.); 2 Laboratorio HLA, Instituto Nacional de Enfermedades Respiratorias Ismael Cosío Villegas, Mexico City 14080, Mexico; rfalfanv@iner.gob.mx; 3 Sección de Estudios de Posgrado e Investigación, Escuela Superior de Medicina, Instituto Politécnico Nacional, Mexico City 11340, Mexico; 4 Hospital General de Zacatecas “Luz González Cosío”, Zacatecas 98160, Mexico; 5 Hospital San Agustín, El Carmen 98610, Mexico; 6 Facultad de Medicina, Universidad Nacional Autónoma de México, Campus Ciudad Universitaria, Mexico City 04510, Mexico; 7 Facultad de Medicina, Universidad Popular Autónoma del Estado de Puebla, Campus Puebla, Puebla 72410, Mexico

**Keywords:** IgG4-related disease, Mikulicz’ disease, autoimmune disease, IgG4-RD, salivary gland diseases, dacryoadenitis

## Abstract

**Background:** IgG4-related disease is a rare, chronic inflammatory disorder characterized by lymphoplasmacytic infiltration, ‘storiform’ fibrosis, and elevated IgG4 levels in affected tissues. This disease has a broad and heterogeneous clinical spectrum that includes four main phenotypes: pancreatic–hepatobiliary disease, retroperitoneal/aortic fibrosis, head and neck disease, and Mikulicz syndrome. **Case Description**: An 85-year-old male patient with a clinical presentation, which is unusual outside Asia, of IgG4-related disease phenotype Mikulicz syndrome, characterized by bilateral dacryoadenitis, orbital pseudotumor, and no evidence of significant systemic participation. Despite extensive involvement in the orbital and glandular region, the patient did not develop serious organ complications, a behavior rarely documented in the literature. Despite the serum IgG4 levels being normal (<135 mg/dL), the clinical and radiological picture suggested IgG4-RD, emphasizing the need for a biopsy for a definitive diagnosis. Histopathological examination revealed a dense lymphoplasmacytic infiltrate, storiform fibrosis, and more than 40% IgG4-positive cells, confirming the diagnosis. **Results**: Treatment with prednisone was initiated alongside azathioprine for long-term control. Calcium and vitamin D3 supplementation were added to prevent glucocorticoid-induced osteoporosis. Remarkable clinical improvement was observed within 24 h, with progressive orbital and glandular symptoms resolution. Over a year, the patient exhibited complete resolution of the orbital tumors, total recovery of vision, and no relapses. The only sequelae observed were dry eye. **Conclusions**: This case highlights the need to consider IgG4-RD with normal serum IgG4 levels, the importance of histopathology for diagnosis, and the efficacy of steroids as first-line treatment. A multidisciplinary approach is essential for timely treatment.

## 1. Introduction

IgG4-related disease (IgG4-RD) is an immune-mediated condition of low prevalence. It is characterized by the formation of tumor masses in most cases and the potential to cause permanent organ damage and, in cases where it remains untreated, death [1,2]. A significant challenge associated with this disease, initially documented in 2001 by Hamano et al., is its classification as a rare condition with an overall prevalence and incidence that remains uncertain [3,4,5]. The current estimates indicate an annual incidence of approximately 0.28 to 1.08 per 100,000 individuals. However, these figures may underestimate the true impact of the disease due to its recent identification, the significant unknowns associated with it, and its often-insidious clinical course [4,6,7].

The slow progression of IgG4-RD is also one of its most insidious and dangerous characteristics, facilitating the development of parenchymal lesions and irreversible organ damage if not identified promptly [8]. This condition has a predilection for specific organs, which can be grouped into four main phenotypes: pancreatic–hepatobiliary disease (31%), retroperitoneal fibrosis with or without arthritis (24%), disease limited to the head and neck (24%), and one of the rarest, Mikulicz syndrome with and without systemic involvement (22%) [9].

Mikulicz syndrome is characterized by IgG4-positive, dense lymphoplasmacytic infiltrates, storiform fibrosis, and phlebitis obliterans, which may simultaneously or sequentially affect multiple organs. The precise prevalence of these complications varies between different series. However, it has been reported that there is multi-organ involvement in over 60% of IgG4-RD cases, such as autoimmune pancreatitis, retroperitoneal fibrosis, tubulointerstitial nephritis, autoimmune hypophysis, and Riedel’s thyroiditis, with the pancreas being one of the most affected organs [10,11]. This is especially the case when the lacrimal and salivary glands are involved, which suggests increased systemic activity of the disease [12]. This syndrome, historically considered a variant of Sjögren’s syndrome, is associated with salivary, parotid, and lacrimal gland inflammation (the differences between those syndromes can be found in Table S1). However, the latter is affected in less than 4.3% of cases of orbital lymphoproliferative disorders [13,14,15,16]. Table 1 summarizes the primary findings used to establish the diagnosis of Mikulicz syndrome.

The clinical presentation of IgG4-RD is broad and heterogeneous, which presents a diagnostic challenge. It is often the case that physicians from various specialties are the first to encounter these cases, which underscores the necessity for a multidisciplinary approach and a comprehensive understanding of the disease. However, the management of IgG4-RD remains complex due to the limited scientific evidence and the risk of misdiagnosis, as the condition is often confused with neoplastic, inflammatory, or infectious conditions such as syphilis and tuberculosis [6].

The rarity of IgG4-RD, the limited availability of epidemiological data, and the progressive increase in reported cases in Mexico and Latin America underscore the significance of this clinical case, in which we present a patient with IgG4-RD who, despite exhibiting extensive systemic activity, did not develop serious organ complications, a phenomenon rarely documented in the medical literature. This report aims to contribute to the existing medical knowledge by providing valuable information to facilitate a better understanding of and more timely diagnosis and management of this relatively little-known but potentially serious disease. In addition, it includes a one-year clinical follow-up, documenting the patient’s evolution and sustained response to treatment. This level of detail provides valuable data on the long-term management of this rare disease.

## 2. Case Description

**Figure 1:**
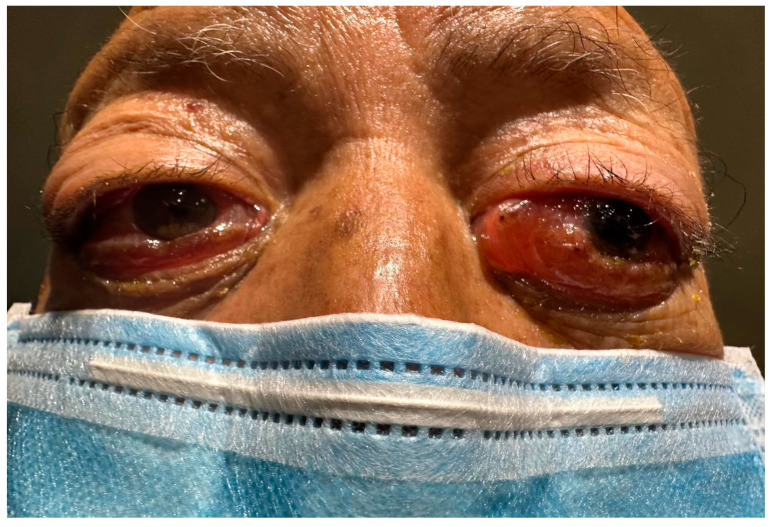
The initial presentation of the patient was a bilateral non-painful mass on the orbital rim.

An 85-year-old male patient with a history of smoking (smoking rate of 55) and exposure to biomass-burning smoke for 40 years was presented for evaluation. No documented previous comorbidities were identified. The patient’s current condition commenced eight weeks before his initial visit, presenting the emergence of a bilateral non-painful mass on the orbital rim. This was accompanied by conjunctival congestion, eyelid edema, and a severe limitation of ocular mobility (Figure 1).

An initial assessment by the ophthalmology department revealed the following ophthalmological history: cataract surgery on the left eye three months earlier, currently without complications. For the ophthalmological examination, the visual acuity of the right eye was recorded as 20/150, while the visual acuity of the left eye was only able to count fingers at three meters. Intraocular pressure was recorded within the normal range at 13 mmHg in both eyes. The Schirmer test range was 3 mm/5 min in both eyes. A palpable bilateral mass was identified on the superior temporal sector of the orbit. It was not painful to palpate.

Ophthalmological evaluation of the anterior segment revealed marked chemosis with areas of conjunctival thickening secondary to exposure, superficial punctate keratopathy, nuclear sclerosis in the right eye, and pseudophakia in the left eye. Fundoscopic examination showed no abnormalities, with no papilledema or signs of retinopathy. Considering these findings, the patient was referred to the infectious diseases department, where they were initiated on a course of antibiotic treatment with carbapenems for five days based on a presumptive diagnosis of periorbital cellulitis. Despite the initial management, the clinical picture showed no significant improvement, which led to the consideration of a more complex underlying process. Consequently, the patient was referred to the rheumatology service for a comprehensive evaluation to rule out an autoimmune disorder as a potential underlying cause.

**Figure 2:**
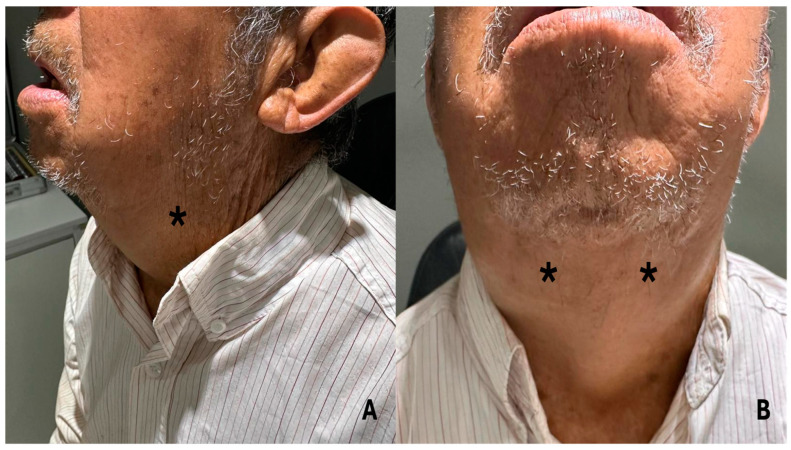
(**A**,**B**): The patient presents with sialoadenitis and parotidomegaly, which impede the ability to close the mouth. * The area of greatest inflammation is indicated on the diagram.

During the evaluation conducted by the rheumatology department, the presence of bilateral exophthalmos was identified, accompanied by the observation of non-painful dacryoadenitis, sialoadenitis, and parotidomegaly (Figure 2A,B). No cardiovascular, pulmonary, or abdominal abnormalities were found on subsequent systemic examination (physical examination, x-ray, and laboratory tests). However, the combination of clinical findings, including ocular involvement, salivary gland involvement, and non-painful masses, indicated that a possible IgG4-related disease might be the underlying condition. This approach highlights the need for a comprehensive assessment in patients with multi-organ involvement to facilitate an accurate and timely diagnosis.

Laboratory studies were conducted and yielded the following pertinent results (normal ranges are described in brackets): The ultra-sensitive C-reactive protein level was 1.29 mg/dL (0.01–0.80), while the immunoglobulin G was 2272 mg/dL (700–1600 mg/dL), immunoglobulin M was 315 mg/dL (40–230 mg/dL), and immunoglobulin E levels were greater than 21,000 UI/mL (<100 UI/mL). Additionally, there was found to be eosinophilia greater than 2000 cells/mm³. Furthermore, liver and pancreatic markers were within the normal range (Table S2). Notably, IgG4 levels remained within the normal range throughout the patient’s follow-up period, fluctuating between 38 and 45 mg/dL. Anti-La/SSB, anti-Ro/SSA, and anti-neutrophil cytoplasmic antibodies (ANCA) led to negative results.

**Figure 3:**
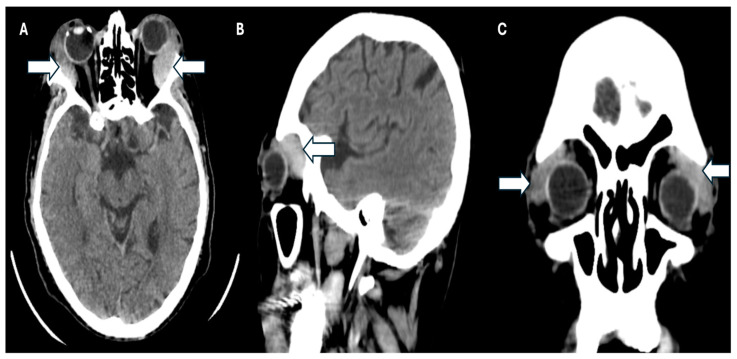
CT scan of the patient. (**A**) Axial plane, (**B**) sagittal, and (**C**) coronal plane where the arrows show infiltration of the adjacent orbital tissue, causing the patient’s bilateral proptosis.

Simultaneously, a simple phase cranial computed axial tomography (CT) scan was requested to continue the approach. This revealed the presence of heterogeneous masses in the upper temporal sector of both orbits at the level of the lacrimal glands and infiltration of the adjacent orbital tissue, causing bilateral proptosis. Additionally, there was evidence of bilateral maxillary sinusitis, with no indication of periorbital cellulitis (Figure 3A–C).

**Figure 4:**
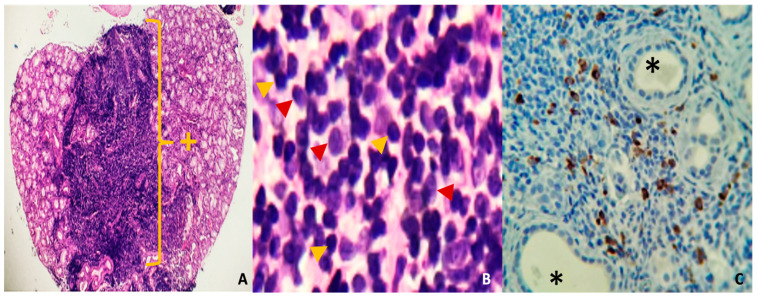
(**A**) (Hye, 40×). Mucinous salivary gland with diffuse lymphoplasmacytic inflammatory infiltrate in the central part of the photomicrograph, replacing acinar and ductal structures. (**B**) (Hye, 400×) dense, mature lymphoplasmacytic infiltrate. The red arrow shows plasma cells, and the yellow arrow shows lymphocytes. (**C**) (IHC, 100×) Mature plasma cells, positive with IgG4 immunostaining (brown). * Salivary gland duct lights +: Area of chronic dense inflammatory infiltrate.

In the absence of elevated IgG4 levels but with a high clinical and radiological suspicion of IgG4-related disease, a biopsy of minor salivary glands was performed to confirm the diagnosis. The biopsy revealed an intense lymphoplasmacytic inflammatory infiltrate with a storiform pattern, and immunohistochemistry showed more than 40% per field of IgG4-positive cells. Furthermore, additional findings are presented in Figure 4A–C. These findings permitted the diagnosis of IgG4-associated disease with the Mikulicz syndrome phenotype without systemic involvement due to IgG4-associated dacryoadenitis, sialoadenitis, and parotidomegaly.

## 3. Results

**Figure 5:**
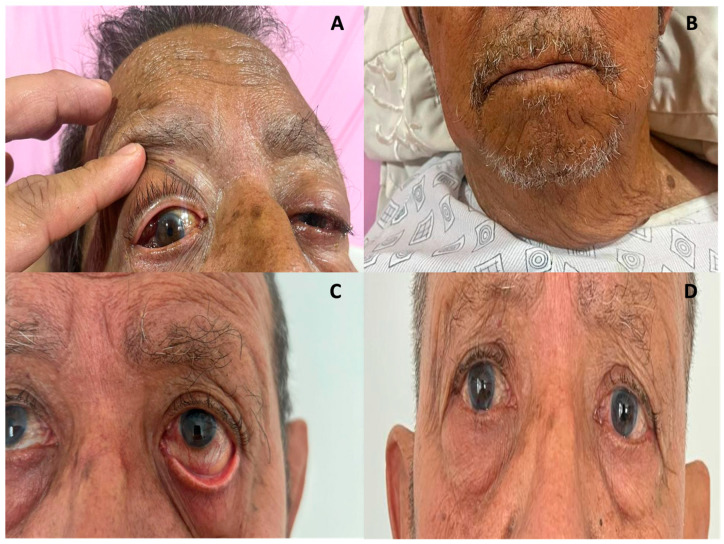
(**A**,**B**): The patient’s clinical response within 24 h of treatment, with a significant decrease in sialoadenitis, dacryoadenitis, and parathyroidomegaly. Patient follow-up at 3- and 12-months post-treatment ((**C**,**D**), respectively).

Following diagnosis, treatment with azathioprine 100 mg/day was promptly initiated in conjunction with the primary treatment regimen comprising prednisone 30 mg/day for one week, followed by a gradual tapering of 5 mg per week up to a maintenance dose of 5 mg per day two months after starting treatment. Calcium and vitamin D3 supplementation were employed to mitigate the risk of glucocorticoid-induced osteoporosis. The clinical response was evident within the first 24 h (Figure 5A,B), with a progressive resolution of the orbital and glandular symptoms.

Subsequent follow-ups were conducted quarterly for one year, during which the patient exhibited a notable clinical remission. This was evidenced by the complete resolution of the orbital mass, accompanied by mild gingival swelling and controlled dry eye symptoms, which were effectively managed with artificial tears (Figure 5C,D). Furthermore, following one year, the patient exhibited an enhancement in visual acuity, with a progression from 20/150 in the right eye and restricted perception in the left eye to 20/25 in both eyes, with no observed limitations in mobility. Additionally, the response rate to IgG4-RD at the one-year follow-up was recorded as 0. To enhance comprehension of the clinical case, a timeline was formulated. This timeline is presented in the Supplementary Materials Figure S1.

## 4. Discussion

IgG4-RD is a rare fibroinflammatory disorder characterized by the development of fibrous tumor-like masses that can affect almost any organ. This report details an exceptional case of Mikulicz syndrome with orbital and glandular manifestations in an 85-year-old male patient. Despite a prolonged period without treatment, the patient did not develop significant organ complications. This case study underscores the diagnostic challenges associated with IgG4-RD. It emphasizes the significance of a multidisciplinary approach to enhance the efficacy of early diagnosis, not delay the initiation of effective treatment, and mitigate patient complications and sequelae.

Geographical and epidemiological context adds to the uniqueness of this case. This clinical report originates from Latin America, where the literature on IgG4-RD is extremely limited. This case significantly contributes to the global understanding of the disease by presenting clinical data from an underrepresented population. The epidemiology of IgG4-RD remains poorly described outside of Asia, particularly regarding the phenotype presented in this clinical case. Most of the available data originate from Japan, where a higher incidence is observed in women with an average age of 55 years and a diagnostic interval of 2–3 years from symptom onset. [17,18] This case, however, involves a man over 85 years of age, which is inconsistent with the predominant epidemiological features and illustrates the limitations in our current understanding of this disease.

Following this, it has been documented in small cohorts that the majority of patients have a history of occupational exposure to solvents, gases, and petroleum products. This suggests the possibility of chronic antigenic stimulation of environmental triggers in the immunopathogenesis of the disease [4].

In this case, the patient had a significant history of smoking and exposure to wood smoke for more than 40 years, which is consistent with the hypotheses of chronic antigenic stimulation and underscores the necessity for further investigation of these factors.

Another notable point is the patient’s atypical clinical presentation. Despite the extensive orbital and glandular involvement observed in imaging studies, the patient did not develop severe organ complications, which are rarely described in the literature. This observation broadens our understanding of the clinical heterogeneity of IgG4-RD and its progression. Although IgG4 is the least abundant IgG subclass (representing less than 5% of total IgG in healthy individuals), elevated serum levels of this subclass are frequently associated with disease activity [19]. However, up to 40% of patients with biopsy-confirmed IgG4-RD have normal IgG4 levels, but a clinical picture and imaging data suggestive of IgG4-RD (Table 1) [20]. This phenomenon occurred in our patient, highlighting the need for a comprehensive evaluation encompassing a detailed clinical history, thorough physical examinations, advanced imaging techniques (such as CT scans), and histopathological studies [9].

In non-Asian populations, such as Caucasians, IgG4-RD is inclined to manifest with a predilection for pancreatic–hepatobiliary involvement, as well as a retroperitoneal and aortic disease; however, these phenomena were absent in our patient. This finding is of particular significance as it illustrates that IgG4-RD can manifest with atypical phenotypic patterns, emphasizing the necessity for a personalized clinical approach to prevent delayed or inaccurate diagnoses.

Early diagnosis and a multidisciplinary approach further distinguish this case. The diagnosis was achieved within eight weeks, allowing treatment to begin before irreversible complications developed. This underscores the importance of an interdisciplinary strategy and a high index of clinical suspicion to ensure timely identification and management of similar cases. Furthermore, the case demonstrates the absence of systemic complications despite a prolonged period without treatment, which not only adds diagnostic complexity but also reinforces the variability in the clinical behavior of the disease. This rare phenotype highlights the necessity for meticulous examination of clinical indicators, particularly in cases where the patient does not align with the conventional demographic or clinical characteristics associated with IgG4-RD. This approach could prove instrumental in identifying atypical presentations and expanding the knowledge base surrounding this rare and complex disease [9].

It is similarly crucial to underscore the necessity of a multidisciplinary methodology for diagnosing IgG4-RD, mainly when clinical manifestations are uncommon. The patient displayed clinical manifestations and radiological findings consistent with IgG4-RD despite normal serum IgG4 levels. Considering this discrepancy, a minor salivary gland biopsy was conducted, ultimately corroborating the diagnosis. This case illustrates the continued importance of histopathological studies as the gold standard for identifying this disease, mainly when serological tests and imaging are inconclusive, and for differentiating this disease from other conditions such as neoplasms, infectious processes, or inflammatory diseases. [6] This approach aligns with international guidelines and serves as a practical example for clinicians working in resource-limited areas where IgG4-RD may be underdiagnosed.

A histological examination should be performed whenever possible, as it remains the basis for a definitive diagnosis [6]. The interpretation of histopathological findings is of paramount importance in the management of IgG4-RD. The diagnostic criteria include the presence of a dense lymphoplasmacytic-type inflammatory infiltrate comprising a significant number of IgG4-positive plasma cells, fibrosis with a distinctive ’storiform’ pattern (resembling the spokes of a cartwheel), obliterative phlebitis and mild to moderate tissue eosinophilia [17]. The biopsy results revealed the presence of a lymphoplasmacytic inflammatory infiltrate with a distinctive storiform pattern, accompanied by a plasma cell count exceeding 40% of IgG4-positive cells, as confirmed by immunohistochemical analysis. These findings permitted the establishment of a definitive diagnosis of IgG4-RD with Mikulicz syndrome phenotype without evidence of systemic involvement.

The treatment of IgG4-RD is primarily concerned with the induction of remission, relapse prevention, and organ function preservation. Glucocorticoids are the initial therapeutic mainstay due to their rapid efficacy in most patients. In this case, the early initiation of glucocorticoids resulted in a marked clinical improvement within 24 h, thereby underscoring the effectiveness of this strategy in controlling active inflammation and reducing the acute manifestations of the disease. Our report also provides valuable insights through its long-term follow-up. The one-year clinical follow-up documented the patient’s sustained response to treatment, offering critical data on managing this rare disease and demonstrating the low relapse rate with this treatment and excellent follow-up.

It has been demonstrated that glucocorticoid therapy results in a notable reduction in the number of follicular helper T cells (Tfh), plasmablasts, and CD4+ cytotoxic T lymphocytes (CD4+ CTLs), which contributes to the resolution of fibroproliferative inflammation. However, the long-term management of IgG4-RD remains a significant challenge, given that it is a chronic disease with a high relapse rate, particularly if glucocorticoids are not supplemented with immunomodulatory agents during the maintenance phase [21].

In patients who experience frequent relapses or intolerance to glucocorticoids, immunosuppressive agents such as azathioprine, mycophenolate mofetil, and methotrexate have been employed as therapeutic options. Furthermore, the use of rituximab, an anti-CD20 monoclonal antibody, has been demonstrated to be an efficacious approach for inducing remission in patients who are refractory to or contraindicated for glucocorticoids. Furthermore, recent studies indicate that rituximab may have a role in long-term relapse prevention, particularly in patients with high levels of persistent plasmablasts [22].

The prognosis of patients with IgG4-RD is contingent upon early diagnosis and appropriate treatment. While the disease responds rapidly to glucocorticoids, delayed diagnosis can result in progressive fibrosis and irreversible organ damage, which has a detrimental impact on patients’ quality of life. In this case, the absence of systemic complications despite a prolonged period without treatment demonstrates the heterogeneity in the clinical course of the disease and its therapeutic response. This case also exemplifies the value of adhering to established diagnostic and therapeutic criteria. It provides a practical example for clinicians, particularly in resource-limited settings, where IgG4-RD is frequently underdiagnosed or misinterpreted.

Conversely, relapses present a substantial challenge in managing IgG4-RD, with up to 50% of patients experiencing recurrences. Clinical monitoring and assessment by biomarkers such as serum IgG4 and plasmablast levels must be conducted with regular imaging studies to detect residual disease activity and facilitate timely adjustments to the treatment plan.

This case study demonstrates the efficacy of glucocorticoid therapy in the initial resolution of symptoms and the necessity of developing tailored strategies for follow-up and relapse prevention in each patient. Furthermore, it highlights the necessity for continued research in IgG4-RD to optimize therapeutic options and define evidence-based protocols to improve long-term outcomes in this rare and complex disease.

The lack of familiarity with IgG4-related disease presents a significant challenge in diagnosis. It is, therefore, vital to maintain efforts to raise awareness of this disease among medical professionals, ensuring that it is recognized and treated on time.

## 5. Conclusions

This case highlights the importance of considering IgG4-RD even when serum IgG4 levels are normal, as clinical symptoms and imaging findings may suggest this diagnosis. Histopathological findings are crucial to confirm the diagnosis, highlighting the value of biopsy as the gold standard for definitive diagnosis. Furthermore, this case reaffirms that steroids should be the first-line treatment as they significantly improve the patient within the first 24 h. Finally, we emphasize the importance of a multidisciplinary team providing early and comprehensive treatment.

## Figures and Tables

**Table 1:** A diagnosis of IgG4-RD-associated Mikulicz syndrome is made based on the following clinical criteria [17].

1. Clinical - The presence of bilateral painless, asymmetric lacrimal, parotid, and submandibular gland tumors. - Elevated risk of developing autoimmune pancreatitis, retroperitoneal fibrosis, tubulointerstitial nephritis, autoimmune hypophysis, and Riedel’s thyroiditis. 2. Hematological - Elevated serum IgG4 levels at more than 135 mg/dL. - Peripheral eosinophilia. - Polyclonal hypergammaglobulinemia. - Elevated IgE. 3. Imaging - A CT scan will show diffuse or focal swelling of organs or soft tissue masses with soft tissue attenuation, well-defined margins, and homogeneous enhancement in the late stage. 4. Histopathological features - A dense lymphoplasmacytic inflammatory infiltrate with increased numbers of IgG4+ plasma cells and often increased numbers of eosinophils. - A storiform pattern of fibrosis. - Infiltration of IgG4+ plasma cells (More than 30 cells per field).

## Data Availability

The data presented in this study are available on request from the corresponding author. The data are not publicly available due to privacy.

## Supplementary Materials

The following supporting information can be downloaded at: https://www.mdpi.com/article/10.3390/jcm14030958/s1, Figure S1: Timeline showing the clinical course of the disease up to one-year follow-up; Table S1: Differences between IgG4-associated Mikulicz syndrome and Sjögren syndrome; Table S2: Laboratory results of the patient on arrival at the rheumatology department.

## References

[1] Bledsoe J.R., Della-Torre E., Rovati L., Deshpande V. (2018). IgG4-related disease: Review of the histopathologic features, differential diagnosis, and therapeutic approach. APMIS.

[2] Della-Torre E., Lanzillotta M., Doglioni C. (2015). Immunology of IgG4-related disease. Clin. Exp. Immunol..

[3] Uchida K., Masamune A., Shimosegawa T., Okazaki K. (2012). Prevalence of IgG4-Related Disease in Japan Based on Nationwide Survey in 2009. Int. J. Rheumatol..

[4] Katz G., Stone J.H. (2022). Clinical Perspectives on IgG4-Related Disease and Its Classification. Annu. Rev. Med..

[5] Hamano H., Kawa S., Horiuchi A., Unno H., Furuya N., Akamatsu T., Fukushima M., Nikaido T., Nakayama K., Usuda N. (2001). High serum IgG4 concentrations in patients with sclerosing pancreatitis. N. Engl. J. Med..

[6] Lanzillotta M., Mancuso G., Della-Torre E. (2020). Advances in the diagnosis and management of IgG4 related disease. BMJ.

[7] Umehara H., Okazaki K., Masaki Y., Kawano M., Yamamoto M., Saeki T., Matsui S., Sumida T., Mimori T., Tanaka Y. (2012). A novel clinical entity, IgG4-related disease (IgG4RD): General concept and details. Mod. Rheumatol..

[8] Katz G., Hernandez-Barco Y., Palumbo D., Guy T.V., Dong L., Perugino C.A. (2024). Proliferative features of IgG4-related disease. Lancet Rheumatol..

[9] Wallace Z.S., Zhang Y., Perugino C.A., Naden R., Choi H.K., Stone J.H., ACR/EULAR IgG4-RD Classification Criteria Committee (2019). Clinical phenotypes of IgG4-related disease: An analysis of two international cross-sectional cohorts. Ann. Rheum. Dis..

[10] Yamamoto M., Takahashi H., Ohara M., Suzuki C., Naishiro Y., Yamamoto H., Shinomura Y., Imai K. (2006). A new conceptualization for Mikulicz’s disease as an IgG4-related plasmacytic disease. Mod. Rheumatol..

[11] Yu W.-K., Tsai C.-C., Kao S.-C., Liu C.J.-L. (2018). Immunoglobulin G4-related ophthalmic disease. Taiwan J. Ophthalmol..

[12] Yamamoto M., Takano K., Kamekura R., Suzuki C., Ichimiya S., Himi T., Nakase H., Takahashi H. (2018). Stage classification of IgG4-related dacryoadenitis and sialadenitis by the serum cytokine environment. Mod. Rheumatol..

[13] Japanese Study Group of IgG4-Related Ophthalmic Disease (2013). A prevalence study of IgG4-related ophthalmic disease in Japan. Jpn. J. Ophthalmol..

[14] Yamamoto M., Harada S., Ohara M., Suzuki C., Naishiro Y., Yamamoto H., Takahashi H., Imai K. (2005). Clinical and pathological differences between Mikulicz’s disease and Sjögren’s syndrome. Rheumatology.

[15] Yamamoto M., Takahashi H., Naishiro Y., Isshiki H., Ohara M., Suzuki C., Yamamoto H., Kokai Y., Himi T., Imai K. (2008). Mikulicz’s disease and systemic IgG4-related plasmacytic syndrome (SIPS). Jpn. J. Clin. Immunol..

[16] Maslinska M., Kostyra-Grabczak K. (2024). Immunoglobulin G4 in primary Sjögren’s syndrome and IgG4-related disease—Connections and dissimilarities. Front. Immunol..

[17] Floreani A., Okazaki K., Uchida K., Gershwin M.E. (2021). IgG4-related disease: Changing epidemiology and new thoughts on a multisystem disease. J. Transl. Autoimmun..

[18] Stone J.H., Zen Y., Deshpande V. (2012). IgG4-related disease. N. Engl. J. Med..

[19] Aalberse R.C., Stapel S.O., Schuurman J., Rispens T. (2009). Immunoglobulin G4: An odd antibody. Clin. Exp. Allergy.

[20] Deshpande V., Zen Y., Chan J.K., Yi E.E., Sato Y., Yoshino T., Klöppel G., Heathcote J.G., Khosroshahi A., Ferry J. (2012). Consensus statement on the pathology of IgG4-related disease. Mod. Pathol..

[21] Kamisawa T., Okazaki K. (2017). Diagnosis and Treatment of IgG4-Related Disease. Curr. Top Microbiol. Immunol..

[22] Khosroshahi A., Wallace Z.S., Crowe J.L., Akamizu T., Azumi A., Carruthers M.N., Chari S.T., Della-Torre E., Frulloni L., Goto H. (2015). International Consensus Guidance Statement on the Management and Treatment of IgG4-Related Disease. Arthritis Rheumatol..

