# Supplementary material for: IgG4-RD-Associated Mikulicz Syndrome Without Classic Systemic Involvement—A Case Report
Source: J Clin Med. 2025 Feb 2;14(3):958. doi: 10.3390/jcm14030958 (PMC11818687; doi:10.3390/jcm14030958)
Supplement: Supplementary file 1 [file jcm-14-00958-s001.zip › jcm-3401537-supplementary.pdf]

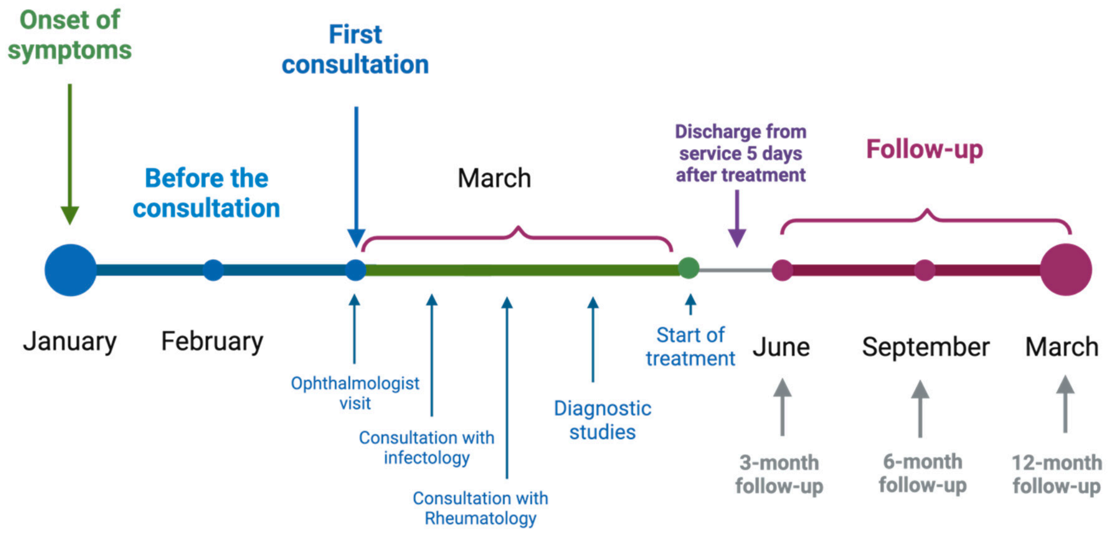

Supplementary Figure S1. Timeline showing the clinical course of the disease up to one-year follow-up.

Supplementary Table S1. Differences between IgG4-associated Mikulicz syndrome and Sjögren syndrome [16]

IgG4 related disease	Sjögren syndrome
- M>F<br>- Dacryoadenitis<br>- Sialadenitis<br>- - +/- inflammation and enlargement of tear and salivary glands<br>- Serum IgG4 concentration is usually elevated<br>- Dry mouth +/-<br>- Associated autoantibodies none<br>- Histopathological findings associated with the presence of IgG4<br>- Other systemic manifestations: interstitial lung disease, tubulointerstitial nephritis, pancreatic involvement, Riedel's thyroiditis	- F>M<br>- Dacryoadenitis<br>- Sialadenitis<br>- There may be inflammation of salivary glands with a risk of lymphoma<br>- Normal or low serum IgG4 concentrations<br>- Dry mouth++<br>- Presence of Anti-La/SSB, anti-Ro/SSA, and ANCA positives<br>- Histopathological findings with the presence of germinal centers, lymphoplasmacytic infiltrate<br>- Other systemic manifestations: tubulointerstitial nephritis, interstitial lung disease, primary biliary cirrhosis, Hashimoto's thyroiditis
F: Female. M: Male. ANCA: Anti-neutrophil cytoplasmic antibodies	

Supplementary Table S2. Laboratory results of the patient on arrival at the rheumatology department

**Liver function tests**	**Normal range**
- AST: 10 U/L	- <32 U/L
- ALT: 14.75 U/L	- <33 U/L
- Total bilirubin: 0.15 mg/dL	- < 1.2 mg/dL
- GGT: 8 U/L	- 9-75 U/L
**Renal tests**	
- Urea: 26 mg/dL	- 16.6-48.5 mg/dL
- Creatinine 0.9 mg/dL	- 0.5 – 1.2 mg/dL
**Pancreatic tests**	
- Amylase: 30 U/L	- 40 – 140 U/L
- Lipase: 35 U/L	- 10 - 140U/L
